# Changes in renal parameters and their association with subclinical vector-borne infections in Bernese Mountain dogs

**DOI:** 10.1186/s12917-020-02506-0

**Published:** 2020-08-12

**Authors:** C. Preyß-Jägeler, K. Hartmann, R. Dorsch

**Affiliations:** Clinic of Small Animal Medicine, LMU University of Munich, Veterinaerstrasse 13, 80539 Munich, Germany

**Keywords:** Proteinuria, Canine, Lyme disease, Anaplasmosis, Glomerulonephritis, Chronic kidney disease, Azotemia

## Abstract

**Background:**

An increased risk for glomerulonephritis and a higher prevalence of antibodies to *Borrelia (B.) burgdorferi* sensu lato have been reported in Bernese mountain dogs (BMDs). The aim of the study was to determine the prevalence of laboratory abnormalities suggestive of kidney disease in clinically healthy BMDs compared to a control population and to investigate if there is a correlation with the occurrence of antibodies to *B. burgdorferi* sensu lato, *Ehrlichia* canis, and *Anaplasma (A.)* spp. and with the occurrence of *Dirofilaria (D.) immitis* antigen*.*

A total of 197 BMDs and 57 control dogs were included in the study. Laboratory evidence of kidney disease was defined as renal azotemia and/or proteinuria with a urine protein creatinine ration of more than 0.5 in an inactive urine sediment. A SNAP®4Dx® ELISA (IDEXX, Laboratories, Inc., Westbrook, ME, USA) was used to detect antibodies to *B. burgdorferi* sensu lato, *E. canis* and *Anaplasma* spp. and antigen of *D. immitis*.

**Results:**

Laboratory evidence of kidney disease was significantly more common in BMDs than in control dogs (17.8% versus 1.8%) (*p* = 0.005). The proportion of BMDs with anti-*B. burgdorferi* sensu latu antibodies and anti-*A. phagocytophilum* antibodies was significantly higher in BMDs (*p* <  0.001). However, an association between these findings could not be identified.

**Conclusion:**

BMDs are more often affected by kidney disease and have a higher prevalence of antibodies to bacterial pathogens transmitted by Ixodes ticks than control dogs. However, a causal relationship between these two variables could not be established due to a lack of association between these two findings.

## Background

Chronic kidney disease is a common cause of morbidity and mortality in dogs [1]. Some studies showed that renal disease is one of the most frequent causes of death in Bernese mountain dogs and among the diseases associated with the shortest median survival time in this breed [2, 3]. In previous studies, glomerular diseases have been shown to occur more often in Bernese mountain dogs (BMDs) than in other breeds [4–8]. An increased incidence of glomerulonephritis in BMDs was first described in 1991 in a doctoral thesis at the University of Zurich [9]. In this five-year retrospective study and a following twenty-four months prospective study [9], frequency, etiology, and laboratory parameters of kidney diseases in BMDs were investigated. The histological changes in the kidneys in dogs presented a complex picture of glomerular changes including membranous, mesangioproliferative, membranoproliferative, and chronic-sclerosing types. A genetic cause could neither be supported nor refuted through a pedigree analysis [9]. Subsequent studies described a BMD typical membranoproliferative glomerulonephritis (MPGN) comorbid with interstitial nephritis [7, 8]. Based on pedigree analysis, there was a high probability that the susceptibility for MPGN in BMDs has a hereditary cause [7]. Furthermore, the same study demonstrated the presence of IgG immunofluorescence assay (IFA) antibodies to *Borrelia (B.) burgdorferi* sensu lato in all investigated BMDs. However, immunohistochemical examination for that organism in sections of the myocardium and kidney was negative in all dogs. Therefore, this study and other subsequent studies could not prove a causal relationship between the presence of antibodies to *B. burgdorferi* sensu lato and glomerular disease [7–13].

Several studies revealed a significantly higher prevalence of antibodies to *B. burgdorferi* sensu lato and to *Anaplasma (A.) phagocytophilum* in BMDs than in control dogs indicating a higher infection prevalence [14, 15]. Chronic persistent infections, such as with *B. burgdorferi* sensu lato, *Ehrlichia* spp., and *Anaplasma* spp. as well as *Dirofilaria spp.* are potential causes of glomerular disease [16]. Despite the fact that an association of *Borrelia* spp. infection and the presence of kidney disease has not convincingly been proven in dogs, in the United States some authors describe a disease entity in Labrador and Golden Retrievers as “Lyme nephritis” and it was speculated whether this disease would be similar to what is seen in BMD in Europe [17, 18].

So far, there are no large studies investigating the prevalence of laboratory abnormalities suggestive for kidney disease in BMDs in comparison to an age- and weight-matched control group. It is also not clear if the higher prevalence of *Anaplasma* spp. antibodies is associated with a higher prevalence of laboratory abnormalities indicating the presence of kidney disease.

The aim of the study was to determine the prevalence of laboratory abnormalities suggestive of kidney diseases in clinically healthy BMDs compared to a control population. Furthermore, it was investigated whether there is an association between serum biochemical and urinalysis results suggestive of kidney disease and the presence of antibodies to *B. burgdorferi* sensu lato, *Ehrlichia canis*, and *Anaplasma* spp. and of *Dirofilaria (D.) immitis* antigen.

## Results

### Signalment and history

The body weight ranged from 25 to 68 kg (median: 39.6 kg) in BMDs and from 30 to 67 kg in control dogs (median: 39.0 kg) (*p* = 0.437). The proportion of BMDs and control dogs that lived in an urban area was similar (29.9% versus 29.8%; *p* = 0.986). There were no significant differences between BMDs and control dogs with regard to the number of walks per week in a forest or grassland (*p* = 0.660), tick exposure (*p* = 0.728), or routine tick prevention (*p* = 0.241). Likewise, other questionnaire-related information did not differ significantly between BMDs and control dogs (Table 1).

**Table 1:** Signalment and history of all dogs included in the study

	Bernese mountain dogs (*n* = 197)	Control dogs (*n* = 57)	P
**Gender**			
Female	126/197 (64.0%)	29/57 (50.9%)	0.085
Male	71/197 (36.0%)	28/57 (49.1%)	
**age (years)** **mean, range**	3.9 (0.5–13)	4.5 (1–12)	0.079
**weight (kilograms)** **(mean, range)**	39.64 (25–68)	38.96 (30–67)	0.491
**Environment**			
Urban	59/197 (29.9%)	17/57 (29.8%)	0.986
Rural	138/197 (70.1%)	40/57 (70.2%)	
**Number of walks per week in grassland or forest**	< 1: 6/197 (3.0%)	< 1: 4/57 (7.0%)	0.684
	1–3: 67/197 (34.0%)	1–3: 17/57 (29.8%)	
	> 3: 124/197 (62.9%)	> 3: 36/57 (63.2%)	
**tick exposure**			
Never	17/197 (8.6%)	4/57 (7.0%)	0.728
Rare	57/197 (28.9%)	16/57 (28.1%)	
Occasionally	71/197 (36.0%)	27/57 (47.4%)	
Frequent	52/197 (26.4%)	10/57 (17.5%)	
**Regular tick prevention (every 6 weeks)**	126/197 (64.0%)	32/57 (56.1%)	0.298

### Laboratory findings

Haematocrit and the concentration of total protein did not differ significantly between BMDs and control dogs. However, BMDs had significantly higher albumin, creatinine, urea and phosphorus concentrations than the control dogs (Table 2). Results of urinalysis are illustrated in Table 3. There were no differences in the results of urine dipstick, urine sediment and urine specific gravity (USG) between groups. Eight BMDs and one control dog were excluded from the determination of urine protein creatinine ratio (UPC) because microscopic examination of urine sediment showed signs of inflammation. The UPC of BMDs was significantly higher than that of control dogs (*p* <  0.001). Proteinuria with a UPC ≥ 0.5 (range 0.57–6.39, median 1.1) was seen in 27/189 (14.3%) BMDs and 1/56 (1.8%) control dogs (UPC 0.78) (*p* = 0.008). Ten of the proteinuric BMDs had a UPC ≥ 2.

**Table 2:** Results of haematology and serum biochemistry of Bernese mountain dogs and control dogs

Variable (unit)	Bernese mountain dogs *n =* 197		Control dogs (*n =* 57)		P
	Mean	SD	Mean	SD	
Haematocrit (l/l)	48.13	+/− 5.448	48.32	+/− 5.932	0.643
WBC (k/μl)	8.47	+/−2.429	7.99	+/−3.363	0.171
Urea (nmol/l)	9.61	+/− 5.522	6.85	+/− 1.831	**< 0.001**
Creatinine (μmol/l)	115.70	+/− 61.487	82.30	+/− 16.727	**< 0.001**
Total protein (g/l)	66.17	+/− 6.613	64.84	+/− 5.902	0.245
Albumin (g/l)	36.46	+/− 3.044	33.97	+/− 4.563	**< 0.001**
Phosphorus (mmol/l)	1.50	+/− 0.501	1.25	+/− 0.291	**< 0.001**

bold = significant difference

*SD* standard deviation, *WBC* white blood count

**Table 3:** Urinalysis results of Bernese Mountain dogs and control dogs

Variable	Bernese mountain dogs (*n =* 197)	Control dogs (*n =* 57)	*p*-value
USG^a,^ (median, range)	1.030 (1.004–1.050)	1.029 (1.008–1.048)	0.684
Protein^b^ (median, range)	+ (neg - +++)	Neg (neg - ++)	0.108
pH^b^ (median, range)	6 (5–9)	6 (5–8)	0.277
Bilirubin ^b^ (median, range)	Neg (neg – ++)	Neg (neg – +)	0.204
Glucose^b^ (median, range)	Neg (neg – 2)	Neg (neg – 2)	0.566
Blood^b^	Neg (neg – ++++)	Neg (neg – +++)	0.163
WBCs/ hpf (median, range)	Neg (neg – 22)	Neg (neg – 22)	0.906
UPC mean +/− SD	(*n =* 189)	(*n* = 56)	
	0.32 +/− 0.853	0.08 +/− 0.127	**< 0.001**

bold = significant difference

*neg* negative, *SD* standard deviation

^a^ Urine specific gravity was determined by a hand refractometer

^b^ Protein, bilirubin, pH, glucose, blood were analysed by dipstick analysis

^c^ Urine protein and creatinine were measured with an automated analyser

Renal azotemia (creatinine > 125 μmol/l and USG <  1.030) was diagnosed in 35/197 (17.8%) BMDs. Twenty-seven of 197 (13.7%) BMDs had renal azotemia and were also proteinuric (UPC > 0.5). In ten of these BMDs, the UPC was ≥2.0 (5.1%). Among the control dogs, 1/57 (1.6%) had renal azotemia. This dogs was proteinuric as well. The proportion of dogs with evidence of kidney disease was significantly higher in BMDs than in control dogs (*p* = 0.005). Laboratory evidence of kidney disease was significantly more common in male (77.1%; 27/35) than in female BMDs (22.9%; 8/35) (*p* <  0.005). BMDs with evidence of kidney disease (mean 5.2 years +/− 2.46) were significantly older than BMDs without evidence of kidney disease (mean 3.7 years +/− 2.65) (*p* = 0.002).

### Prevalence of antibodies to *B. burgdorferi* sensu lato, *Ehrlichia canis*, and *Anaplasma* spp. and of *Dirofilaria* immitis antigen

Antibodies to *B. burgdorferi* sensu lato attributable to infection were detected in 44.6% of BMDs (88/197) and in 21.1% of control dogs (12/57) (*p* = 0.001), and 46.2% of BMDs (91/197) and 19.3% of control dogs (11/57) had anti-*Anaplasma* spp. antibodies (*p* <  0.001). There was a significant difference in the prevalence of anti-*B. burgdorferi* sensu latu antibodies (*p =* 0.001) (OR 3.03) and anti- *Anaplasma* spp. antibodies (*p <*  0.001) (OR 3.59) between BMDs and control dogs. There was no significant association between the sex of BMDs and and the presence of antibodies against *B. burgdorferi* sensu latu (*p* = 0.181), *Anaplasma* spp. (*p* = 0.371), or the presence of both pathogens (*p* = 0.594).

Antibodies to *B. burgdorferi* sensu latu were present in 54.3% of BMDs (19/35) with evidence of kidney disease and in 42.6% of BMDs (69/162) without evidence of kidney disease (*p* = 0.209). Antibodies to *Anaplasma* spp. were detected in 34.3% of BMDs (12/35) with evidence of kidney disease and 48.8% of BMDs (79/162) without evidence of kidney disease (*p* = 0.120)*.* Antibodies to both pathogens, *B. burgdorferi* sensu latu and *Anaplasma* spp. were detected in 22.3% (44/197) of BMDs compared to 1.8% (1/57) of the control dogs (*p* <  0.001) with no significant difference between BMDs with and without evidence of kidney disease (11.4% (4/35) vs. 24.1% (40/162); *p* = 0.088). Regarding proteinuria alone, there was no association between the presence of proteinuria and the presence of antibodies against these pathogens, neither the group of BMDs (*p =* 0,118) nor in the control group (*p* = 0.404).

Of all dogs included in the study, only one BMD (1/267) was positive for *D. immitis* antigen*.* This dog hat renal azotemia (creatinine 167 μmol/ l, USG 1.015) and was proteinuric with a UPC of 2.79. No dog in the study had antibodies to *Ehrlichia canis* (Table 4).

**Table 4:** Prevalence of antibodies and signalement in Bernese Mountain dogs (BMDs) with and without laboratory evidence of kidney disease

	BMDs without evidence of kidney disease (*n* = 162)	BMDs with evidence of kidney disease (*n* = 35)	BMDs with UPC ≥ 2 (*n* = 10)	P
age (years) mean, range	3.7 (1–13)	5.2 (2–8)	5.1 (1–11)	0.652
Female	117/162	9/35	1/10	**< 0.001**
Male	45/162	26/35	9/10	**< 0.001**
Positive for *Borrelia burgdorferi* antibodies	69/162 (42.6%)	19/35 (54.3%)	6/10 (60.0%)	0.288
Positive for *Anaplasma* spp. antibodies	79/162 (48.7%)	12/35 (34.3%)	2/10 (20.0%)	0.079
Positive for antibodies against *Anaplasma* spp. and *Borrelia burgdorferi*	40/162 (24.7%)	4/35 (11.4%)	1/10 (10.0%)	0.148
Positive for antigen of *Dirofilaria immitis*	0/162 (0%)	1/35 (2.9%)	1/10 (10.0%)	0.334
Positive for antibodies against *Ehrlichia canis*	0/162 (0%)	0/35 (0%)	0/10 (0%)	

bold = significant difference

## Discussion

In the present study, 17.8% of the BMDs had laboratory evidence of kidney disease compared to 1.6% of the control dogs. Interestingly, renal azotemia was the most common abnormality and was more common than proteinuria in this population of BMDs, and none of the BMDs had proteinuria without azotemia. Persistent proteinuria quantified by measuring UPC in a urine sample with an inactive urine sediment is the hallmark of glomerular disease and is the only laboratory abnormality present in early stages of this disease [16, 19]. However, all dogs with azotemia without proteinuria had mild azotemia with creatinine values between 126 μmol/l to 174 μmol/l and, even though the USG was below 1.030, none of these dogs was isosthenuric. Not being fasted and higher muscle mass are possible reasons for increased creatinine as well, however, this would also apply for the control dogs. Determination of symmetric dimethylarginin (SDMA) would have helped to exclude this influence but was not available when the study was performed [20]. Therefore, it is possible that some of the dogs would not have been classified as dogs with renal disease if repeated measurements of creatinine and USG or measurement of glomerular filtration rate (GFR) were performed to confirm the presence of renal disease. A UPC of > 0.5 is abnormal for a dog but there is no clear cut off for UPC that is diagnostic for any specific renal disease because the overlap in ranges is too broad to be clinically reliable [19]. However, there is a consensus that with increasing magnitude of proteinuria assessed by UPC, there is also increased likelihood for primary glomerular disease [19, 21]. While renal proteinuria with UPC values between 0.5 and 2.0 can be due to tubulointerstitial disease, glomerular disease, or a combination of both, UPC ≥ 2, which was observed in ten dogs in the present study, is sufficiently high to suggest the presence of pathological glomerular proteinuria and therefore primary glomerular disease [19]. These ten dogs in the present study had UPC values between 2.1 and 6.4, they were all azotemic, and had a USG <  1.020 [7, 8]. Interestingly, that even though all these dogs had a USG < 1.020, owners did not recognize their dogs as being polydipsic and polyuric. To the owners and upon physical examination these dogs appeared healthy, but regarding the relevant laboratory parameters, these ten dogs resemble dogs of previous reports on familial MPGN in BMDs that had more pronounced proteinuria (UPC 4–30) and azotemia as well [7, 8]. Seventeen of the 35 dogs with renal azotemia had a UPC between 0.5 and 2, which can be due to tubulointerstitial disease, glomerular disease, or a combination. It is possible that these dogs were in an early stage of MPGN and had additional tubulointerstitial nephritis with less pronounced laboratory abnormalities or a different disease entity.

In the present study, 77.1% (27/35) and 90.0% (9/10) of dogs with laboratory evidence of kidney disease and evidence of glomerulopathy, respectively, were male. This is different from the 1:4 ratio of males to females that has been reported for BMDs with familiar MPGN [7, 8] but similar to the gender distribution of a study performed in Switzerland with 160 BMDs [22]. In that study, 11/160 dogs (6.7%) had evidence of kidney disease defined as UPC > 0.3 in an inactive urine sediment [22]. In the Swiss study, three of these eleven BMDs with UPC > 0.3 had mild renal azotemia. While only 28.1% (45/160) of the total study population were male dogs, 82% (9/11) of dogs with evidence of kidney disease were male [22]. In the present study as well, male BMDs (27/35, 77.1%) were significantly more likely to have laboratory evidence of kidney disease than female BMDs (8/35, 22.9%) despite female BMDs being over-represented in the overall study population (126/197; 63.6%). This gender distribution could indicate that BMDs might be predisposed for other forms of renal disease that affect male individuals with greater frequency.

The average age of BMDs with evidence of kidney disease in the present study was 5.2 years. The mean age of dogs with evidence of glomerular disease was 5.1 years with no difference between dogs with UPC > 2 and those with UPC between 0.5 and < 2 or renal azotemia only. Compared to a study in the UK in which only around 10% of dogs with CKD were younger than 7 years [23], dogs in the present study were affected at a young age. This suggests that there is not only a breed predisposition for glomerular but also for tubulointerstitial kidney disease in BMDs.

In the present study, there was no correlation between the presence of laboratory abnormalities indicating kidney disease and the presence of antibodies against *B. burgdorferi* and *Anaplasma* spp.. In a previous study including 53 *B. burgdorferi* antibody-positive and -negative BMDs and 30 antibody-positive and -negative control dogs [24], dogs were followed for more than 2 years after they had been tested positive for *B. burgdorferi* antibodies. There were, however, no alterations in laboratory parameters (blood and urine) that would indicate development of renal disease [24].

The higher prevalence of antibodies to *B. burgdorferi* sensu lato and *Anaplasma* spp. in BMDs compared with control dogs indicates a breed predisposition to infection with *B. burgdorferi* sensu lato and *Anaplasma* spp. which is in agreement with the results of other studies [14, 25]. In the present study, the effects of coat colour, hair length, size and living conditions on antibody titers were controlled for by using control dogs that were heavier than 30 kg, had long dark hair and lived in environments similar to those of the BMDs. Therefore, this difference cannot be attributed to environmental factors. A satisfying explanation for the high prevalence of antibodies to *B. burgdorferi* sensu lato and *Anaplasma* spp. in BMDs has not been established*.*

Only one BMD (1/267) of all dogs included in the study was *D. immitis* antigen-positive. This BMD had a UPC > 2, and an infestation with *D. immitis* could be responsible for the proteinuria in this BMD.

The most common biochemical findings that have been reported in dogs with *Anaplasma* spp. and *B. burgdorferi* infections are hyperproteinaemia, hypoalbuminaemia and hypoproteinaemia [18, 26, 27]. In the present study, there was no difference in serum total protein serum albumin between antibody-positive and antibody-negative dogs. The significantly higher albumin in BMDs compared to control dogs did not exceed the reference range and was not considered to be of clinical relevance.

This study has several limitations. Firstly, dogs included in this study were tested only once and thus comparison or confirmation of initial and follow-up results was not possible. To diagnose persistent renal proteinuria, ideally two or more urine samples collected several weeks apart would have been desirable. This is also true for USG and serum creatinine which are influenced by extrarenal factors, such as hydration status and diet, and repeated measurement would be preferable to document persistence of abnormal results [28]. In addition, determination of SDMA would have been particularly helpful to exclude the influence of the muscle mass [29].

Secondly, in the present study, only routine renal functional parameters creatinine, urea, USG and UPC were used for assessment of renal function. More sensitive markers for GFR (SDMA and cystatin C), tubular damage (urinary neutrophil gelatinase-associated lipoprotein (uNGAL), urinary retinol-binding protein (uRBP)), and glomerular damage (urinary immunoglobulin G and urirnary C-reactive protein) were not measured. Urinary NGAL and uRBP have been shown to detect possible acute tubular injury not identified with conventional renal parameters creatinine, urea and USG in dogs with parvovirus enteritis [30], and babesiosis [31]. However, UPC had the capacity to detect acute kidney injury as well. Therefore, it is unlikely that the use of more sensitive markers of kidney injury would have led to markedly different results.

## Conclusions

In conclusion, BMDs had more often laboratory evidence of kidney disease than control dogs*.* Male dogs were affected nearly ten times as often as female dogs (OR 9.6). Furthermore, BMDs had a higher prevalence of antibodies to *B. burgdorferi* sensu lato and *Anaplasma* spp. than control dogs of various breeds. There was no correlation between laboratory parameters indicating renal disease and the presence of antibodies to *B. burgdorferi* sensu lato and *Anaplasma* spp..

## Methods

### Data acquisition

The clinical data and laboratory test results were derived from data collected for another study with prospectively enrolled dogs [15]. Apart from the published results, additional data was collected and additional laboratory tests were performed for the study that have not yet been analyzed. Data analysis for the current study was approved by the ethical committee of the veterinary faculty of the LMU Munich (210–04-04-2020).

### Dogs

A total of 197 purebred BMDs (verified by registry certificates) and 57 control dogs originating from the same areas in Southern Germany were included in the study. For control dogs, the selection criteria for inclusion in the study were a long, dark hair coat and a body weight of more than 30 kg, which is comparable with that of BMDs. All control dogs lived in the same households as the BMDs or in the same neighborhoods. The control group consisted of 29 female dogs (10 spayed) and 28 male dogs (13 neutered) ranging in age from 1 to 12 years (median: 4.5 years).

Bernese Mountain Dogs were located with the help of breeder clubs and consisted of 126 females (27 spayed) and 71 males (18 neutered) ranging in age from 6 months to 13 years (median: 3.9 years).

All dogs underwent a physical examination and blood sampling, and all of the dogs were examined by the same veterinarian (first author). Owners were asked to answer a detailed questionnaire regarding clinical signs, environment, tick exposure, tick prevention, and vaccination history of their dogs.

### Laboratory variables

Haematocrit, white blood cell count (WBC), a partial serum biochemical profile, and urinalysis were performed for all dogs. For the serum biochemical profile, blood samples collected from the jugular or cephalic vein were placed in plastic serum tubes, and then were allowed to clot for 30 min. Samples were centrifuged at 3000 rpm (RPM) for 10 min and the serum was harvested. Serum biochemical analysis was performed using an automated analyser (Hitatchi 911; Roche Deutschland Holding GmbH, Grenzach-Wyhlen, Germany) and included the following parameters: the concentration of urea, creatinine, total protein, albumin, and phosphorus. The Cell Dyn 3500 (Fa. Abbott, Wiesbaden) was used for determination of the haematocrit and the WBC.

Urine samples were collected by cystocentesis in all 254 dogs. A complete urinalysis including determination of the USG, a dipstick analysis, and microscopic examination of the urine sediment was performed. The USG was determined with a refractometer (ATC; Müller GmbH, Erfurt, Germany). For examination of the urine sediment, urine was centrifuged at 1500 RBM for 5 minutes. The urine sediment was assessed for the presence of red blood cells, white blood cells, and bacteria in 20 fields using a 40 x objective lens. UPC was determined from the supernatant using an automated analyser (Hitatchi 911; Roche Deutschland Holding GmbH, Grenzach-Wyhlen, Germany). UPC was not determined in nine dogs because they had more than five leukocytes per field or bacteria in the urine sediment.

Laboratory evidence of kidney disease was defined as renal azotemia (creatinine > 125 μmol/l and USG < 1.030) and/or an increased UPC (UPC ≥ 0.5). Additionally, BMDs with UPC ≥ 2.0 were categorized as a separate group because a UPC ≥ 2 is sufficiently high to suggest the presence of pathological glomerular proteinuria and therefore glomerular disease [19].

### Antibody testing

Whole blood samples were collected from a jugular or cephalic vein in all dogs and tested for anti-*B. burgdorferi* sensu lato, anti-*Anaplasma* spp.*,* anti-*E. canis* antibodies and *Dirofilaria immitis* antigen using the SNAP®4Dx® ELISA (IDEXX, Laboratories, Inc., Westbrook, ME, USA) according to the manufacturer’s instructions. Briefly, each test kit consisted of a coated matrix with a result window containing five spots; colour development in the spots indicated the presence of anti-*B. burgdorferi*, anti-*Anaplasma* spp. and anti-*Ehrlichia canis* antibodies and *Dirofilaria immitis* antigen. The fifth spot served as a positive control. The wash and substrate solutions were placed in two separate chambers, and after activation of the test kit, the solutions flowed across the coated matrix. The test was performed immediately after blood collection using whole blood.

### Statistical analysis

Data were recorded and analysed using the commercial computer program SPSS Statistics version 24.0 (IBM Deutschland GmbH, Munich, Germany). Differences were considered significant at *p* <  0.05. The Chi-square test or Fisher’s exact test were used for investigation of the categorical parameters of the urinalysis and for the investigation of the differences of antibodies and antigen between the different groups. The other laboratory findings of BMDs and controls dogs were compared using the nonparametric Mann Whitney U test. The Mann Whitney U test was also used to analyse differences in laboratory findings between antibody-positive and antibody-negative dogs.

## Data Availability

The datasets used and/or analysed during the current study are available from the corresponding author on reasonable request.

## Abbreviations

*A*: *Anaplasma*
*B*: *Borrelia*
BMD: Bernese Mountain dog
*D*: *Dirofilaria*
GFR: Glomerular filtration rate
IFA: Immunofluorescence assay
MPGM: Membranoproliferative glomerulonephritis
OD: Odds ratio
SD: Standard deviation
SDMA: Determination of symmetric dimethylarginine
UPC: Urine creatinine protein ratio
USG: Urine specific gravity
